# Cases of Malformation of the Eye and Ear—Anophthalmos

**Published:** 1869-03-15

**Authors:** 


### *Cases of Malformation of the Eye and Ear—Anophthalmos.*

REPORTED AT A MEETING OF THE CHICAGO MEDICAL SOCIETY.

Dr. Holmes reported two cases of congenital absence of both eyes, which had very recently fallen under his observation. The reporter stated that such cases were exceedingly rare, even in the aggregate experience of occulists.

One of the infants, a female, of German parentage, was born January, 1868. The parents were perfectly healthy, as also were two other children. They could ascribe no cause for the abnormal condition of their last child.

With the exception of the absence of the eyes, and an abnormally narrow palpebral tissue, the child, as far as could be observed, was strong and perfect. When it was at rest, one could scarcely remark any thing unusual in its appearance. The brows were very full, the cheeks high. The orbits were not large enough to fairly receive the tip of the fore-finger. On passing two probes into either orbit and separating them, the edges of the lids, not more than the fifth of an inch in length, could be seen at the depth of nearly half-an-inch, in the small, conical cavity of the orbit. Between the lids mucous membrane could be seen, but no trace of a globe except in the left orbit, in which was a minute, reddish elevation behind the lids.

The child grew well till July, when it was attacked with cholera infantum, and died after a few weeks.

The second case, also a female, under the care of Dr. H. M. Lyman, presented almost precisely the same appearances. There was, however, a malformation of the nose, in two respects. There was a slight triangular deficiency of the outer wall of the right nostril, near the tip of the nose. The nose was not placed exactly in the median line of the face, but obliquely towards the left side. There was, also, a peculiar ridge extending from the nose obliquely upward and to the left, over the left os frontis. In other respects the child, a first-born, was perfect. The parents, American, could give no cause, even imaginary, of the deformity, *congenital absence of the external meatus.*

Dr. Holmes also reported two cases of congenital absence of the external meatus, which he had observed during the last three weeks.

A girl, eight years and three months old, of German parentage, was brought for advice regarding her hearing.

She was pale, and presented a very peculiar expression of the face, the head being exceedingly thin; the external wall of each orbit being deficient far toward the ear

There was also a deficiency of the orbital border of the superior maxillary bone, of the malar bone and zygoma. The lower jaw was somewhat receding; the teeth were irregular, but not notched. The roof of the mouth was abnormally arched. The right ear presented the following peculiarities: There were, externally, simply the lobe and mere rudiments of the upper portion of the pinna, and of the tragus and anti-tragus. There was not a trace of an external meatus.

The left ear presented precisely the same appearances, although the rudiments of the external ear were somewhat differently arranged.

The patient could understand loud conversation, but was so diffident that it was impossible to determine the distance at which she could hear a watch. The hearing was improved by opening the mouth.

In less than three weeks after this patient consulted Dr. H., a German lithographer, twenty-three years of age, sought advice in reference to his myopia. He remarked during the examination, that he had no right ear. There was the same condition as in the case just described. The pinna, however, was somewhat more extensively developed. The hearing was more deficient than in the preceding case. He could not hear a watch placed over his ear.

Dr. Holmes also reported the case of a little girl, one year old, in which there seems to be a congenital paralysis, or possibly an absence of the levator muscle of each upper lid. The palpebral fissure is abnormally short, the globes themselves being remarkably small. The lids appear almost absolutely closed. No known injury occurred to the child, either at or after its birth. She is healthy and vigorous, with no other peculiarity in her formation. Vision is acute, as shown by the manner in which the child sees and seizes minute objects.

The head is invariably thrown back whenever the child attempts to look at any thing. This seems to cause the lower lid to fall a line or more from the upper lid. As there is scarcely the slightest motion to the upper lids, it is doubtful how much benefit could be gained by surgical treatment, without danger of inflammations from exposure of the cornea to the action of dust and the atmosphere.

### EXOPHTHALMOS.

Dr. H. recalled attention to the patient with enormous exophthalmos whom he presented to the society some time since, stating that he now has, under his care, a patient afflicted with the same form of congenital disease.

The eyes were also enormously protruded from the orbit; the right one to such an extent that the lids, though greatly hypertrophied and distended, no longer covered the cornea. The motions of this eye were much impeded; those of the other eye nearly normal.

In consequence of an injury of the right cornea, and long-continued exposure to the atmosphere, the vision was already destroyed. There was great pain in the eye, with sympathetic irritation of the cornea in the other eye, to relieve which symptoms the right eye was extirpated.

The refraction and accommodation of the eyes had always been normal.

After the wound caused by the extirpation healed, the lids remained nearly as prominent as if closed over a normal eye.

The patient stated that his father, brother and sister, were born with the eyes abnormally prominent, although the brother's eyes became especially affected at the age of fifteen. The appearance of the brother's would naturally suggest the thought that they were suffering from Graves' (Basedows') disease. There were, however, no evidences of anæmia, or of disease of the heart or thyroid gland.

